# A Short Peptide of Autotransporter Ata Is a Promising Protective Antigen for Vaccination Against *Acinetobacter baumannii*

**DOI:** 10.3389/fimmu.2022.884555

**Published:** 2022-04-13

**Authors:** Peng Sun, Xin Li, Chao Pan, Zhicheng Liu, Jun Wu, Hengliang Wang, Li Zhu

**Affiliations:** ^1^ State Key Laboratory of Pathogen and Biosecurity, Beijing Institute of Biotechnology, Beijing, China; ^2^ School of Medicine, Tsinghua University, Beijing, China; ^3^ State Key Laboratory of Biochemical Engineering, Institute of Process Engineering, Chinese Academy of Sciences, Beijing, China

**Keywords:** *Acinetobacter baumannii*, *acinetobacter* trimeric autotransporter adhesin (Ata), CpG, CTB, subunit vaccine

## Abstract

With the emergence of multidrug-resistant strains, *Acinetobacter baumannii* infection is becoming a thorny health problem in hospitals. However, there are no licensed vaccines against *A. baumannii*. *Acinetobacter* trimeric autotransporter (Ata) is an important known virulence factor located on the outer membrane of bacteria. Herein, we carried out a series of experiments to test the immunogenicity of a short C-terminal extracellular region of Ata (Ata_α_, only containing 39 amino acids) in a murine model. The short peptide Ata_α_ was fused with the cholera toxin B subunit (CTB), which has been reported to have immunoadjuvant activity. The fusion protein showed no inflammation and organ damages, and have the ability to elicit both Th1 and Th2 immune responses in mice. The bactericidal activities against *A. baumannii* and prophylactic effects of the fusion protein were further evidenced by a significant reduction in the bacterial load in the organs and blood. In addition, the candidate vaccine could provide broad protection against lethal challenges with a variety of *A. baumannii* strains. Moreover, when CpG was added on the basis of aluminum adjuvant, the immune response, especially cellular immunity, could be further strengthened. Overall, these results revealed that the Ata_α_ is a promising vaccine target against *A. baumannii* infection.

## Introduction

*Acinetobacter baumannii*, a Gram-negative bacterium, is an opportunistic pathogen that usually causes nosocomial infections (1, 2) through wound sites and mechanical ventilation (3, 4), resulting in high mortality rates (5, 6), especially in the ICU, where it can reach 40% (6). Although the use of antibiotics facilitates the treatment of bacterial infection, the bacterium has developed multiple mechanisms of resistance to antibiotics, such as regulation of antibiotic transport through bacterial membranes, mutational alteration or enzymatic modification of antibiotic target (7). In 2017, the World Health Organization released a list of 12 “global priority pathogens,” in which carbapenem-resistant *A. baumannii* was classified as “critical” grade (8).

Vaccination is one of the most cost-effective strategies to control the infection of multidrug-resistant *A. baumannii*. At present there are no licensed *A. baumannii* vaccines on the market. Whole-cell vaccines and outer membrane vesicle vaccines have been proven to induce strong immune responses in murine models (9–12). However, safety concerns related to the complexity of components, incomplete inactivation, and the presence of LPS were often raised (13). Subsequent studies have focused on single-component vaccines (e.g., subunit vaccines). Some promising antigens, such as OmpA, OmpW, OMP22, FilF, BamA, and Bap, have been proven to generate protective effects (14–19). In addition, the capsular polysaccharide (CPS) of *A. baumannii* could also be used as effective antigens for the development of vaccines. However, the diversity of the CPS structure (over 100 unique capsule loci) (20, 21) impedes the development of CPS-based vaccines.

*Acinetobacter* trimeric autotransporter (Ata) of *A. baumannii* ATCC 17978, belongs to the trimeric autotransporter adhesin superfamily and is secreted through a type V secretion system. Trimeric autotransporter adhesins act as key virulence factors in many Gram-negative bacteria; participate in adhesion, biofilm formation, immune evasion, angiogenesis, and cell death; and mediate adhesion to extracellular matrix proteins in a murine pneumonia model (8, 22, 23). Ata is a potential vaccine target, and research has shown that antibodies against Ata are highly opsonic against *A. baumannii* and show low-to-moderate killing activity against four *A. baumannii* strains (22, 24). Further, a 263-amino acid conserved fragment from the C-terminus of Ata was confirmed to elicit a specific antibody response and provide protection against the challenge in mice. The immunized sera also reduced the formation of biofilm and the adherence of *A. baumannii* (25). This evidence suggests that the C-terminal fragment from Ata is a promising target for the development of an *A. baumannii* vaccine. However, as a subunit vaccine, the low immunogenicity of the antigen requires immune enhancement strategies, such as the introduction of delivery systems or the use of adjuvants, to achieve an effective immune response. In the delivery system of prophylactic vaccines, proteinaceous carriers are ideal because of their high safety and biocompatibility. However, proteinaceous antigens (especially large antigens) are prone to interfere with the carrier resulting in protein misfolding and structural instability. Therefore, a shorter epitope is beneficial for the further design of the vaccine.

The B subunit of cholera toxin (CTB), which forms a pentameric ring structure, is widely used as a delivery vehicle and adjuvant for various antigens (26–30). Studies have shown that the coupling of CTB with antigens can stimulate specific Th1 and Th2 immune responses. In general, the CTB pentamer can bind to GM1, which is widely distributed on the surface of B cells and dendritic cells.Thus, CTB-based vaccines offer advantages in vaccine delivery, endocytosis, and antigen presentation. However, steric hindrance between CTB and the antigen might disrupt the formation of pentamers, suggesting that the selection of a smaller antigen could benefit the formation of pentamers and antigen presentation (31).

In this study, we used only 39 amino acids of Ata as an antigen and evaluated its immunogenicity by coupling with CTB. After confirming its safety, a series of animal experiments were carried out, and they indicated that both Th1 and Th2 immune responses were induced and efficient protective effects were demonstrated through lethal and non-lethal infection models. In particular, we found that when CpG was added with aluminum adjuvant, both cellular immunity and humoral immunity could be further strengthened, among which the cellular immunity response was more enhanced. Overall, our results reveal that the candidate antigen, prepared based on 39 amino acids of Ata, is a promising protective target for vaccination against *A. baumannii* infection.

## Materials and Methods

### Bacterial Strains, Plasmids, and Growth Conditions

*Escherichia coli* DH5α was used to clone plasmids, and BL21(DE3) was used for the expression of the recombinant protein CTB-Ata_α_. *A. baumannii* ATCC 17978 was purchased from ATCC, and the clinical isolates XH733 and MDR-ZJ06 were kindly provided by professor Yunsong Yu (Department of Infectious Diseases, Sir Run Run Shaw Hospital, College of Medicine, Zhejiang University). All of the *E. coli* and *A. baumannii* strains were grown in Luria–Bertani broth or on solid medium containing 1.5% agar. The Ata_α_ peptide was fused to the C-terminus of CTB (GenBank: X76390.1) with a flexible linker (GGSG). The full-length CTB-Ata_α_ coding sequence was codon-optimized, synthesized by Sangon Biotech (Shanghai) Co., Ltd., and inserted into pET30a between NdeI and XhoI. pGEX4T-ata was constructed to produce GST-Ata_α_, which was used in the ELISA assay.

### Expression, Renaturation, Purification, and Qualification of CTB-Ata_α_

*E. coli* BL21(DE3)/pET30a-CTB-Ata_α_ cells were cultured in a shake flask at 37°C to an OD_600_ of 0.6. CTB-Ata_α_ expression was induced by 0.5 mM IPTG at 30°C for 12 h. Cells were collected by centrifugation, resuspended by 20 mM Tris-HCl (pH 8.5), and disrupted through a homogenizer with four passes at 700 bar. The CTB-Ata_α_ inclusion bodies were solubilized by lysis buffer (8 M urea and 5 mM DTT), diluted with refolding buffer (20 mM Tris (pH 8.5), 1 mM GSSG, and 1 mM GSH), and purified by anion-exchange chromatography and size-exclusion chromatography. The recombinant CTB-Ata_α_ was analyzed by RP-HPLC (Agilent ZORBAX 300SB-C8, 5 μm, φ4.6 × 250 mm) with a gradient of 0 to 100% elution buffer (95% acetonitrile 0.1% TFA) in 40 min and SEC-HPLC (TSK gel G2000SWXL, 5 μm, φ7.8 × 300 mm) with PBS.

### GM1 Binding Assay

A 96-well plate was coated with 100 μL GM1 solution (2 μg/mL) overnight at 4°C. After washing three times, 300 μL of 5% skim milk was added to each well. After incubation at 37°C for 1 h and washing three times again, samples with different dilutions from 3.3 nM were added and incubated at 37°C for 1 h. After another washing step, 100 μL of anti-CTB antibody (Sigma, Germany) was added to the plate, followed by incubation at 37°C for another hour. Then, the plate was washed again three times, 100 μL of HRP-labeled goat anti-rabbit antibody (Sigma, Germany) was added, and the plate was incubated at 37°C for 1 h. At last, after washing the plate three times, 100 μL of TMB solution (Solarbio, China) was added to the plate, followed by adding 50 μL of 2 M H_2_SO_4_ to stop the reaction, and the dual-wavelength detection was performed at 450 nm and 630 nm.

### Immunization

Six-week-old female BALB/c mice (SPF) were purchased from Beijing Vital River Laboratory Animal Technology Co., Ltd. All of the animal experiments and procedures were performed in accordance with the guidelines of the Academy of Military Medical Sciences Institutional Animal Care and Use Committee (Ethics Approval Code IACUC-DWZX-2020-027). Mice were randomly grouped and for the evaluation of immune effects, and mice were immunized three times at an interval of 2 weeks. Two weeks after the second and third immunizations, blood was collected and the serum was separated for further analysis.

### ELISA

First, 96-well plates were coated with GST-Ata_α_ (10 μg/mL, 100 μL/well) overnight at 4°C, washed three times with PBST (PBS with 0.05% Tween 20), and blocked with 300 μL of 5% skim milk in PBST at 37°C for 2 h. Next, the wells were incubated with 100 μL 3-fold serially diluted serum from 1:50 at 37°C for 1 h. After another washing step, 100 μL of HRP-conjugated goat anti-mouse IgG, IgG1, IgG2a, or IgG2b (Beijing Biodragon Immunotechnologies Co., Ltd.) was added to each well, and plates were incubated at 37°C for 1 h, followed by washing five times with PBST, and then 100 μL of TMB solution (Solarbio Life Sciences, China) was added to each well. The reactions were stopped with 50 μL of 2 M H_2_SO_4_, and the dual-wavelength detection was performed at 450 nm and 630 nm.

### Opsonophagocytic Killing Assay

The opsonophagocytic assay was performed as previously described (32). Briefly, HL60 cells (ATCC, CCL-240) were cultured in RPMI1640 medium containing 10% heat-inactivated fetal calf serum. After 3 weeks of continuous culture, HL60 cells (6 × 10^5^ cells/mL) were differentiated in RPMI1640 containing 0.8% N N-dimethylformamide (Sigma, Germany) for 4 days. HL60 cells (4 × 10^5^ cells per well), *A. baumannii* ATCC 17978 (1 × 10^3^ CFUs per well), and complement (Pel-Freez, USA) were added into the wells along with the heat-treated mouse serum (56°C for 30 min for the inactivation of endogenous complement components). After 45 min incubation at 37°C in 5% CO_2_, the microtiter plates were placed on ice for 20 min to terminate the reaction. Finally, the mixtures were diluted and plated in duplicate on LB plates for bacterial counting. Serum killing rates were calculated by comparing the number of CFU with naïve serum samples.

### Flow Cytometry

For cell surface marker staining, draining lymph nodes (dLNs) of each mouse were individually collected and triturated into a single cell suspension. Then the cells were stained with different combinations of flow cytometry antibodies for 30 min at 4°C, which included APC-conjugated anti-mouse CD3 (eBioscience, USA), eFluor450-conjugated anti-mouse CD4 (eBioscience, USA), PE-conjugated anti-mouse CD8a (eBioscience, USA), APC-conjugated anti-mouse B220 (Biolegend, USA), Pacific Blue-conjugated anti-mouse GL-7 (Biolegend, USA), PE-conjugated anti-mouse CD95 (Biolegend, USA), FITC-conjugated anti-mouse CD4 (Biolegend, USA), PE-conjugated anti-mouse PD-1 (Biolegend, USA), and Brilliant Violet 421-conjugated anti-mouse CXCR5 (Biolegend, USA). After washing with staining buffer (eBioscience, USA), the cells were dispersed in 500 μL of staining buffer (eBioscience, USA) and analyzed by flow cytometry (Beckman Coulter, Cytoflex LX).

### Determination of Bacterial Loads, Cytokine Concentration, and Survival Rate

To evaluate the bacterial load, *A. baumannii* ATCC 17978 was cultured at 37°C to an OD_600_ of about 2.0 and then diluted with saline to approximately 2.0 × 10^7^ CFU/200 µL. At 12 h after intraperitoneal inoculation, blood (10 µL) was collected and mixed with saline (990 µL), and organs (spleen and lungs) were removed, homogenized with saline (1 mL), and then collected in 2.0-mL microcentrifuge tubes as initial samples. After sample was placed at 4°C for 15 min, the supernatant of each initial sample was diluted and cultured on solid LB medium. The bacterial colonies were counted after culturing overnight at 37°C. Blood samples were collected, and the serum levels of IL-1β, TNF-α, and IL-6 were determined using ELISA kits (Dakewe). ATCC strains (ATCC 17978) and clinical isolates (MDR-ZJ06 and XH733) were used to evaluate the survival rates. Each mouse was intraperitoneally injected 14 days after the third immunization with 4.9 × 10^7^ CFU/200 µL of *A. baumannii* ATCC 17978, 1.0 × 10^7^ CFU/200 µL of MDR-ZJ06, and 4.5 × 10^7^ CFU/200 µL of XH733. Survival rates were monitored continuously for 7 days.

### Statistical Analysis

GraphPad Prism 8 was used for statistical analysis. The data are presented as mean ± SD. Data were analyzed by one-way ANOVA with Dunn’s multiple-comparison test for the multiple group comparison. Differences were considered statistically significant at *P* < 0.05 (****P < 0.0001, ***P < 0.001, **P < 0.01, *P < 0.05).

## Result

### Expression, Purification, and Characterization of CTB-Ata_α_

**Figure 1:**
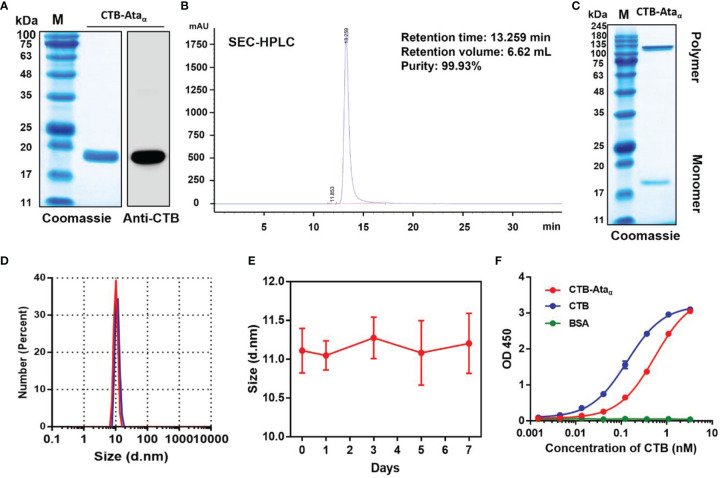
Preparation and characterization of CTB-Ata_α_. **(A)** Identification of purified CTB-Ata_α_ by SDS-PAGE and Western blot with anti-CTB. **(B)** SEC-HPLC (TSK gel G2000SWXL, 5 μm, φ7.8 × 300 mm) to determine the purity of CTB-Ata_α_. **(C, D)** Purified CTB-Ata_α_ was analyzed by non-reducing SDS-PAGE **(C)** and DLS **(D)**. **(E)** DLS analysis of CTB-Ata_α_ size stability at different time points upon incubation at 37°C. Data are presented as means ± s.d. **(F)** The GM1 binding assay was performed to detect the binding activity between CTB-Ata_α_ and GM1. The plate was coated with GM1 at 2 μg/mL (100 μL/well), and commercial CTB and purified CTB-Ata_α_ were 3-fold serially diluted from 3.3 nM. Data are presented as means ± s.d.

Ata are expressed in a variety of *A. baumannii* strains, and its extracellular translocator domain forms an α-helical structure. The peptide consisting of 39 amino acids (Ata_α_), which used as a candidate antigen in this study, acts as a neck between the β-barrel membrane-inserted anchor and the surface-exposed passenger domains (22) (**Supplementary Figure 1**). To confirm the conservation of Ata_α_, 1393 Ata protein sequences of *A. baumannii* were obtained from NCBI database and Ata_α_ was completely consistent with 94% (1303/1393) of the sequences (**Supplementary Figure 2**), indicating a very high conservation of Ata_α_. Although this high conservation gives it the potential to serve as a vaccine target, it is difficult to induce an effective immune response with such a short sequence. Our previous results have revealed that the immunogenicity of antigens could be greatly improved when coupled with CTB. Thus, we investigated whether the specific immune response to Ata_α_ could also be enhanced by the carrier. Firstly, a fusion protein consisting of Ata_α_ and CTB was expressed through an isopropyl-β-D-thiogalactopyranoside (IPTG)-inducible vector, pET30a-CTBAta_α_, in which CTB was fused at the N-terminus of Ata_α_. The recombinant protein CTB-Ata_α_ was highly expressed in the form of inclusion bodies **(** **Supplementary Figure 3**) and refolded by dialyzing against refolding buffer at 4°C overnight. Then CTB-Ata_α_ was obtained through further anion-exchange chromatography and size-exclusion chromatography steps. Purified CTB-Ata_α_ was detected by Coomassie blue staining and confirmed by Western blot with antibodies against CTB (**Figure 1A**). The size-exclusion chromatography results showed that the target protein was eluted in 200-240 mL from a column with a total volume of 450 mL (**Supplementary Figure 4**), indicating the polymer form of the product. The Coomassie blue staining results showed that the purity of CTB-Ata_α_ was very high. Then, SEC-HPLC and RP-HPLC analyses further revealed that the purity of CTB-Ata_α_ was greater than 98% (**Figure 1B** and **Supplementary Figure 5**). In addition, as expected, the retention volume of CTB-Ata_α_ in HPLC was in line with the size-exclusion chromatography results, indicating that CTB may maintain its pentameric state in the fusion protein. In order to further confirm this point, non-reducing electrophoresis was carried out. Coomassie blue staining revealed that the CTB-Ata_α_ band located at a molecular weight of about 100 kDa (**Figure 1C** and **Supplementary Figure 6**). Moreover, dynamic light scattering (DLS) showed a monodisperse sample with a size of 10 nm in diameter (**Figure 1D**). This state can be maintained at 37°C for 7 days (**Figure 1E**). Because the immune enhancement effect of CTB is largely related to the binding ability of GM1, we further analyzed the binding ability of CTB-Ata_α_ to GM1 by ELISA. Although the combination with GM1 was a little decreased compared with natural CTB, it still maintained a strong binding ability for CTB-Ata_α_ (**Figure 1F**). These above results suggested that the fusion protein CTB-Ata_α_ was successfully prepared and maintained pentameric structure and the GM1 binding ability.

### Safety Evaluation of CTB-Ata_α_

**Figure 2:**
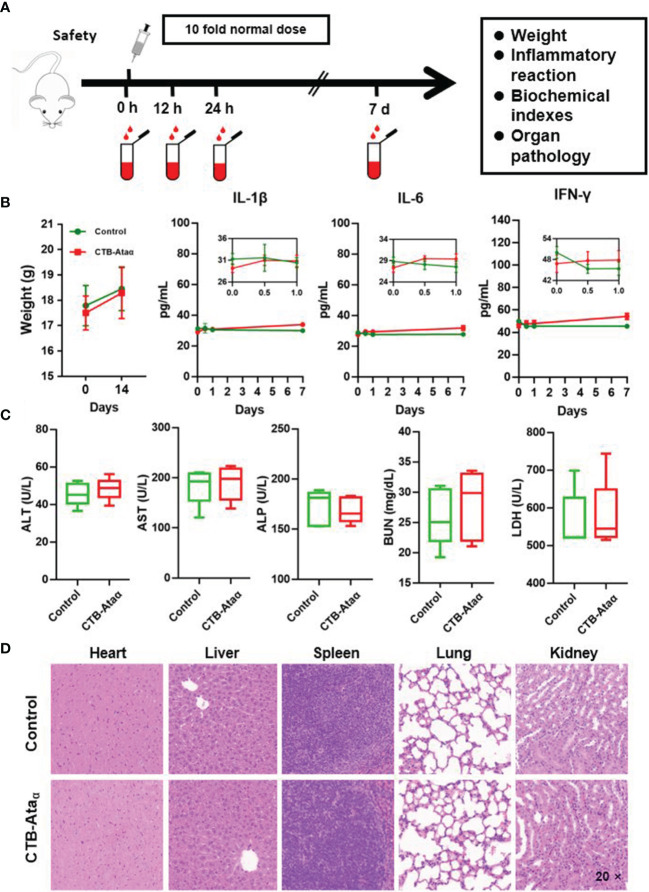
Safety evaluation of CTB-Ata_α_. **(A)** Schematic procedure of the safety estimation. **(B)** Body weight and inflammatory cytokine levels (IL-6, IL-1β, and IFN-γ) in serum at different time points after immunization with 10 times the normal dose. Data are presented as means ± s.d. **(C)** Detection of serum biochemical indices, including ALT, ALP, AST, BUN, and LDH, 14 days after immunization. Data are presented as means ± range. **(D)** HE staining analysis of mouse organs (including heart, liver, spleen, lungs, and kidney) 14 days after immunization.

To evaluate the safety of CTB-Ata_α_, BALB/c mice were immunized with 40 μg CTB-Ata_α_ (10 times the normal dose) and a series of indicators were detected at different time points (**Figure 2A**). Compared with control group (without any treatment), there was no significant difference in average body weight in the CTB-Ata_α_ group (**Figure 2B**). Meanwhile, cytokines, including interleukin-1β (IL-1β), IL-6, and interferon-gamma (IFN-γ), in serum were detected at 0 h, 12 h, 24 h, and 7 days. Their concentrations were within the safe range in both groups, and no significant difference was observed (**Figure 2B**). Because these three indicators reflect the acute inflammatory response, our results indicated that CTB-Ata_α_ has no systemic toxicity. Furthermore, serum biochemical indices were detected 14 days after immunization. All of the indices, including aminotransferase (AST), alanine aminotransferase (ALT), blood urea nitrogen (BUN), lactate dehydrogenase (LDH), and alanine aminotransferase (ALP), were in the normal range **(** **Figure 2C**), indicating the biocompatibility of the vaccine. In addition, HE staining of organ sections showed no organ damage or acute inflammation in the heart, liver, spleen, lung, and kidney 14 days after immunization (**Figure 2D**), which again confirmed the safety of the vaccine.

### Enhancement of the Specific Immune Response of Ata_α_ by Fusing With CTB

**Figure 3:**
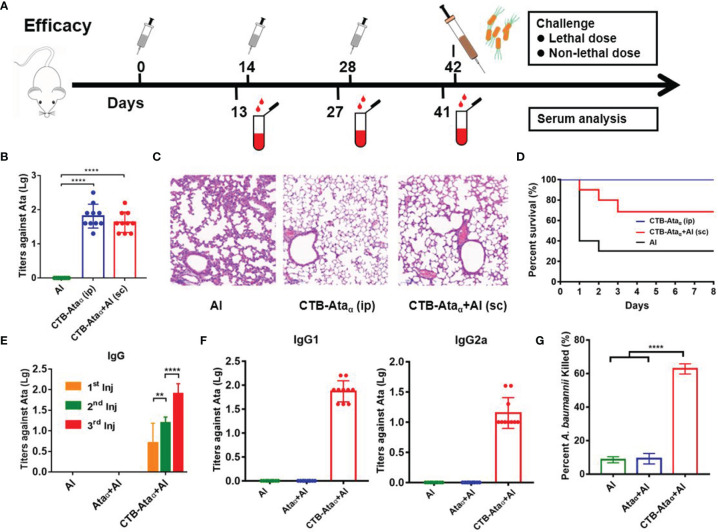
Immune response of CTB-Ata_α_. **(A)** Immunization and efficacy evaluation schedule. **(B)** Comparison of Ata_α_-specific IgG titers between different injection strategies. **(C)** HE staining analysis of lung tissue after infection of immunized mice 12 h post-challenge with a non-lethal dose. **(D)** Survival rates of differently immunized mice. **(E)** Ata_α_-specific IgG levels were measured in the serum of BALB/c mice immunized with Ata_α_+Al and CTB-Ata_α_+Al after immunization. **(F)** Ata_α_-specific IgG subtypes (IgG1 and IgG2a) were detected after the third boost. **(G)** Opsonophagocytic assay. Mouse serum complement, HL60 cells, and (*A*) *baumannii* ATCC 17978 were incubated in round-bottom 96-well plates with shaking and plated in LB medium to measure bacterial survival by counting colony-forming units, and the percentage of killing was calculated. Data are presented as means ± s.d. Each group was compared using one-way ANOVA with Dunnett’s multiple-comparison test. ****P < 0.0001, **P < 0.01.

Having confirmed the safety of the vaccine, we next evaluated the immune effect of Ata_α_fused with CTB. BALB/c mice were immunized three times with an interval of 14 days, and some indices, such as the antibody titers against Ata_α_, the opsonophagocytic activity of immunized serum, and the protection against infection, were evaluated (**Figure 3A**). First, we compared the effects of two commonly used injection methods (subcutaneous and intraperitoneal). The results showed that although the serum antibody titer of intraperitoneally immunized mice was slightly higher than that of subcutaneously immunized mice, there was no significant difference between them (**Figure 3B**). After being challenged with a non-lethal dose (2.0 × 10^7^ CFU/mouse) of *A. baumannii* strain ATCC 17978, the mice were dissected and the bacterial loads in the lung, spleen, and blood were detected. Significant reductions in bacterial load were observed in the lung, spleen, and blood in the two CTB-Ata_α_-immunized groups compared with the control, while there was no significant difference between the CTB-Ata_α_ groups (**Supplementary Figure 7**). Moreover, in the lungs, one of the main target organs of *A. baumannii*, severe tissue injury was observed from control mice, characterized by decreased alveolar expansion, alveolar structure disruption, lung interstitial expansion and inflammatory cell infiltration, while no obvious pathological changes were found in the CTB-Ata_α_ groups (**Figure 3C**). The challenge experiment showed that the survival rates in subcutaneously immunized mice were a little lower (**Figure 3D**). Considering the consistency with the further commercial application of the vaccine, we chose subcutaneous immunization for subsequent studies.

Then, we evaluated the enhancement effect of CTB on the specific antibody response in mice. BALB/c mice were immunized with one of three treatments, Al(OH)_3_ adjuvant (Al), Ata_α_+Al, or CTB-Ata_α_+Al, on days 0, 14, and 28. Blood was sampled on days 13, 27, and 41 to facilitate quantitation of antibodies against Ata_α_. ELISA-based measurement of the IgG titers showed that only CTB-Ata_α_-treated mice showed an Ata_α_-specific antibody response (**Figure 3E**), indicating that the immunogenicity of Ata_α_ was greatly increased by CTB. Further, antibody subtype analysis results showed that the titers of both IgG1 and IgG2a were increased, indicating the enhancement of both specific humoral and cellular immune responses (**Figure 3F**). In addition, to identify the anti-microbial activity of the obtained serum, bactericidal activity was assessed by opsonophagocytic assay. The results indicated that, compared with naïve serum group, bacteria killed by HL60 cells increased to approximately 63% when complement inactivated CTB-Ata_α_ antiserum was present (**Figure 3G**).

### Prophylactic Effects Against *A. baumannii* Infection by CTB-Ata_α_

**Figure 4:**
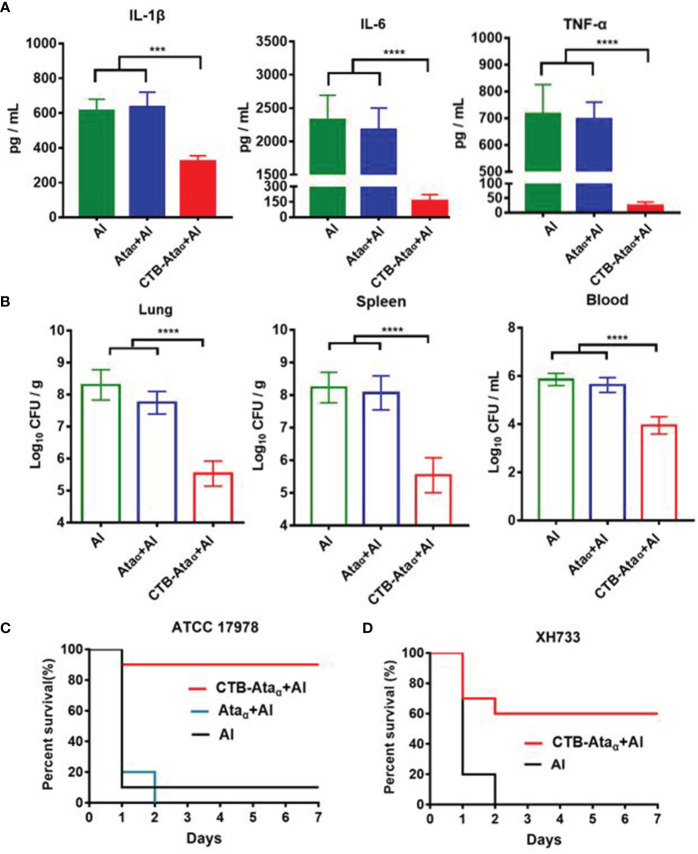
Efficacy evaluation of the CTB-Ata_α_ vaccine. **(A)** Inflammatory cytokine levels 12 h post-challenge with a sublethal dose. **(B)** Bacterial loads of lung, spleen, and blood 12 h post-challenge with a sublethal dose. **(C, D)** Survival rates post-challenge with a lethal dose. ATCC 17978 (4.9 × 10^7^ CFU) and XH733 (4.5 × 10^7^ CFU) were intraperitoneally injected 14 days after the third injection. Data are presented as means ± s.d. Each group was compared using one-way ANOVA with Dunnett’s multiple-comparison test. ****P < 0.0001, ***P < 0.001.

Encouraged by the great enhancement of the antibody response by CTB-Ata_α_, we next evaluated the protective effects of the vaccine against bacterial infection. All of the immunized mice were challenged intraperitoneally with a non-lethal dose of *A. baumannii* strain ATCC 17978 (2.0 × 10^7^ CFU/mouse) 14 days after the third immunization. The blood of mice was collected 12 h after infection and the inflammatory factors (including IL-1β, IL-6, and TNF-α) in serum were measured (**Figure 4A**). The results showed that the concentrations of the three factors were strongly decreased only in CTB-Ata_α_-treated mice, while Ata_α_ alone showed little effect (**Figure 4A**), indicating that immunization with CTB-Ata_α_ can significantly reduce the systemic inflammatory response caused by *A. baumannii* infection. Correspondingly, the bacterial loads in the lung, spleen, and blood dramatically decreased in CTB-Ata_α_-immunized mice, but not in mice treated with Ata_α_ alone (**Figure 4B**), which showed that the vaccine prepared by fusing CTB with Ata_α_ could well reduce bacterial infection of organs. Further, when the challenge dose was increased to 4.9 × 10^7^ CFU/mouse, we found that the mice from both the Al and the Ata_α_ group were died within two days of pathogen challenge, while 90% of the CTB-Ata_α_-treated mice survived (**Figure 4C**). In addition, we also found that CTB-Ata_α_ could exert protective effects against two other *A. baumannii* clinical isolates (**Figure 4D** and **Supplementary Figure 8**), indicating a promising prospect for clinical application.

### Further Enhancements of the Immune Response and Protection of CTB-Ata_α_ by the Addition of CpG Adjuvant

**Figure 5:**
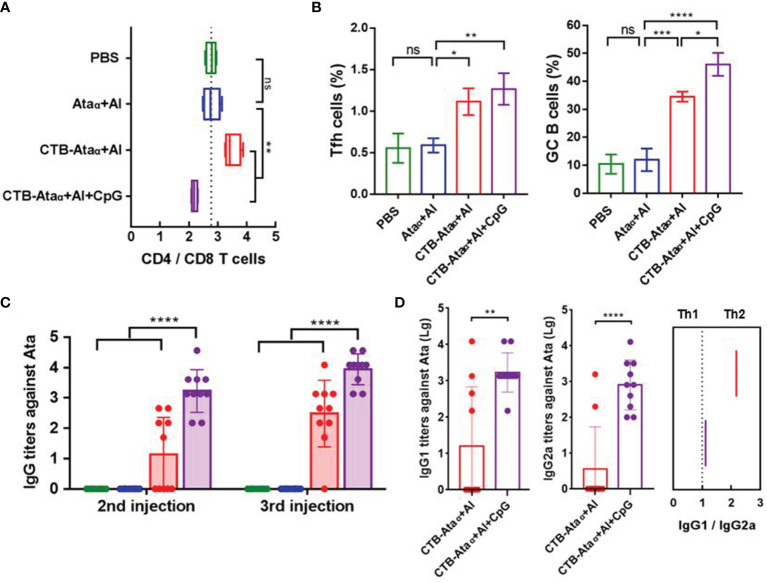
Immunostimulatory effects of CpG. **(A)** The ratio of CD4^+^/CD8^+^ T cells on day 7 after the second injection. **(B)** The proportion of Tfh cells (CXCR5^+^ PD-1^+^ among the CD4^+^ cell population) and GC B cells (GL7^+^ CD95^+^ among the B220^+^ cell population) in dLNs on day 7 postvaccination. **(C)** Ata_α_-specific IgG titers on day 13 postvaccination. **(D)** Ata_α_-specific IgG subtype titers and ratio of IgG1/IgG2a. Data are presented as means ± s.d. Each group was compared using one-way ANOVA with Dunnett’s multiple-comparison test. ****P < 0.0001, ***P < 0.001, **P < 0.01, *P < 0.05; ns, *P* > 0.05.

To further increase the humoral and cellular immune responses, we added CpG adjuvant on the basis of CTB-Ata_α_+Al. As is known, CpG is a TLR9 agonist and has been successfully used as an adjuvant in commercial hepatitis B vaccines (e.g., HEPLISAV-B^®^). To evaluate the immune response types after CpG addition, we analyzed the proportions of CD4^+^ and CD8^+^ T cells from draining lymph nodes (dLNs) 7 days after the secondary immunization according to the previous immunization protocol. We observed significant changes in the ratio of CD4^+^/CD8^+^ T cells between CTB-Ata_α_ + Al and CTB-Ata_α_ + Al + CpG mice (**Figure 5A**). Compared with CTB-Ata_α_ + Al, the CD4^+^/CD8^+^ T cell ratio was decreased significantly in the CTB-Ata_α_ + Al + CpG group, suggesting the enhancement of the cellular immune response. Moreover, the analysis of dLN revealed that the proportions of both T-follicular helper (Tfh) cells and B cells in the germinal center (GC) had increased when CpG was added 7 days after the last immunization (**Figure 5B** and **Supplementary Figure 9**). This result indicated that the addition of CpG also enhances the humoral immune response. Subsequently, we tested the antibody titers of mice after each immunization and found huge increases, consistent with previous results, of total IgG in serum after both the second and the third immunization in CTB-Ata_α_, antibody titers were higher in the CpG group (**Figure 5C**). Through the analysis of two IgG subtypes, we found that the titers of both IgG1 and IgG2a (especially IgG2a) were significantly increased by adding CpG (**Figure 5D**). In addition, the results also suggest that immunization with CTB-Ata_α_ + Al + CpG can produce a higher protection rate than immunization with CTB-Ata_α_ + Al or CTB-Ata_α_ (**Supplementary Figure 10**).

## Discussion

At present, *A. baumannii* infection is a serious concern due to its multiple antibiotic resistance (33), which limits the treatment of infection with multidrug-resistant *A. baumannii* strains. Hence, a safe and effective prophylactic or therapeutic vaccine against this bacterium is urgently needed. In this study, we prepared an *A. baumannii* candidate vaccine by fusing only 39 amino acids of Ata (Ata_α_) with CTB. After confirming the safety of CTB-Ata_α_, a series of animal experiments were performed, which proved that the vaccine can elicit an effective immune response and provide great protection against *A. baumannii* strains, including two clinical isolates, while Ata_α_ alone has no effect. Therefore, our work not only provides a simple and promising *A. baumannii* antigenic peptide, but it also reveals two effective immune enhancement strategies (delivery carrier coupling and adjuvant mixing) for short peptide antigens.

Trimeric autotransporter adhesins, which are secreted through the Type V secretion system and form trimeric complexes, contain an N-terminal signal peptide, a long stalk domain (passenger domain), and a conserved membrane anchoring domain, and are extremely diverse in amino acid length (from hundreds to thousands) (34, 35). Ata, expressed by about 78% of *A. baumannii* clinical isolates, assists in the formation and maintenance of biofilm (22) and adheres to abiotic surfaces (36), human cells, and extracellular matrix components. It may play an important role in immune evasion, probably by binding to host glycan receptors (galactose, N-acetylglucosamine, and galactose (β1−3/4) N-acetylglucosamine) (23). Therefore, Ata was presumed to be an ideal antigen for vaccination, which was verified by several studies (24, 25). In the present study, we focused on the extracellular part of Ata. Although no immune response was detected when Ata_α_ alone, both humoral and cellular immunity can be significantly improved after fusion with CTB. Our results revealed that the antibody against this short peptide is protective, and thus Ata_α_ can be used as a promising antigen for *A. baumannii* vaccine design. It is worth noting that, to our knowledge, Ata_α_, only 39 amino acids, is the shortest reported *A. baumannii* antigen at present. It is compatible with more delivery proteinaceous carriers, avoid misfolding and can be coupled with other epitopes. Moreover, our previous results have shown a simple biological method to prepare an *A. baumannii* polysaccharide conjugate vaccine. By using a protein glycosylation system, *A. baumannii* surface polysaccharide could be coupled with CTB (32). Thus, based on the current results, we can further prepare peptide–polysaccharide dual antigen vaccines in the future.

According to our results, the immune response is greatly improved when the peptide antigen is fused with CTB, suggesting that the carrier plays an important role in the enhancement of antigen immunogenicity. The same effect can be found in conjugate vaccines, which are produced by coupling the weakly immunogenic polysaccharide antigen with carrier protein (37–39). Polysaccharide antigens could be transformed from TI antigen to TD antigen with the help of carriers (40–42). In addition, various vaccine delivery systems have the ability to significantly improve the immune response of antigens (43). Among them, the widely focused nano-sized carriers can not only realize the clustering of antigens, but also efficiently target lymph nodes and present antigens (44). Although the antigen we prepared is too small to be observed by transmission electron microscopy, the size of the vaccine can be improved by coupling Ata_α_ with CTB, and a strong immune enhancement effect was observed. Thus, our next research direction is the development of more efficient delivery vectors to further improve the immune response to Ata_α_. In conclusion, the efficient vaccine delivery system is of great significance to improve the immune effects of antigens, especially weak immunogenic antigens.

CTB has been used as an adjuvant in bacterial and viral vaccines (45–48). It probably directly interacts with antigen presenting cell (49). Many studies have reported that CTB-antigen vaccines stimulate immune responses with a Th2 type profile (50–52). These antigens could be internalized by antigen presenting cells and further presented through MHC II pathways to activate the humoral response. In addition, some researchers have also found that CTB-based vaccines have the capacity to induce a Th1 immune response (29, 53–55), which mainly resulted from the activation of signaling pathways encouraging the antigen presenting cells to secrete Th1 cytokines. Some CTB-based vaccines could induce a polarization of Th1 responses through transcutaneous immunization (53, 56), which might be attributed to the different microenvironment in the regulation of immune responses and dendritic cell heterogeneity between mucosal and non-mucosal tissues. Thus, our results showed that both Th1 and Th2 responses to Ata_α_ were enhanced when fused to CTB and mixed with CpG. Besides, MHC I-restricted antigen presentation plays a key role for CD8^+^ T cell activation. Previous studies have reported CTB has the capacity to enhance MHC I-restricted antigen presentation, due to GM1 binding-dependent cytoplasm transportation (29, 57). We found that CTB still maintained the binding ability with GM1 when fused with Ata, thus promoting the differentiation of CD8^+^ T cells.

Synthetic CpG oligodeoxynucleotides function as pathogen-associated molecular patterns recognized by TLR9, and they are gradually becoming popular adjuvants. CpG could stimulate both innate and adaptive immune responses, and many studies have revealed that CpG has the ability to enhance antigen-specific Th1 biased immune responses (58, 59), and the enhancements of protection against bacteria and viruses have been illustrated in human and animal models (60–63). In this work, the Ata_α_-specific antibody responses could be augmented by fusing Ata_α_ with CTB, while this strengthening effect was further dramatically improved when CpG was added. In particular, the improvement of the cellular immune biased response was greater. This more balanced immune response is conducive to the elimination of pathogens from the body. Consistently, our previous results also revealed that when CpG is combined with aluminum hydroxide adjuvant, the specific immune response could be greatly improved (64). This may be due to the negatively charged CpG which could be better adsorbed by aluminum hydroxide (having a positive charge on the surface), so as to prevent CpG degradation and dilution. Thus, improving compatibility between adjuvants also provides another strategy for the immune promotion of weak immunogenic antigens.

## Data Availability Statement

The original contributions presented in the study are included in the article/**Supplementary Material**. Further inquiries can be directed to the corresponding authors.

## Ethics Statement

The animal study was reviewed and approved by Academy of Military Medical Sciences Institutional Animal Care and Use Committee (Ethics Approval Code IACUC-DWZX-2020-027).

## Author Contributions

Conceptualization and theoretical analysis, JW, HW, and LZ. Methodology, PS, XL, CP, JW, and ZL. Carried out experiments and data analysis, PS, XL, CP, and ZL. Writing—original draft preparation, PS and CP. Writing—review and editing, CP, JW, HW, and LZ. Funding acquisition, CP and LZ. All authors listed have made a substantial, direct, and intellectual contribution to the work and approved it for publication.

## Funding

This work was supported by the National Natural Science Foundation of China (82171819 and U20A20361).

## Conflict of Interest

The authors declare that the research was conducted in the absence of any commercial or financial relationships that could be construed as a potential conflict of interest.

## Publisher’s Note

All claims expressed in this article are solely those of the authors and do not necessarily represent those of their affiliated organizations, or those of the publisher, the editors and the reviewers. Any product that may be evaluated in this article, or claim that may be made by its manufacturer, is not guaranteed or endorsed by the publisher.
