# Supplementary material for: A Short Peptide of Autotransporter Ata Is a Promising Protective Antigen for Vaccination Against Acinetobacter baumannii
Source: Front Immunol. 2022 Apr 13;13:884555. doi: 10.3389/fimmu.2022.884555 (PMC9043751; doi:10.3389/fimmu.2022.884555)
Supplement: Supplementary file 1 [file DataSheet_1.pdf]

Supplementary Material

Supplementary Figures

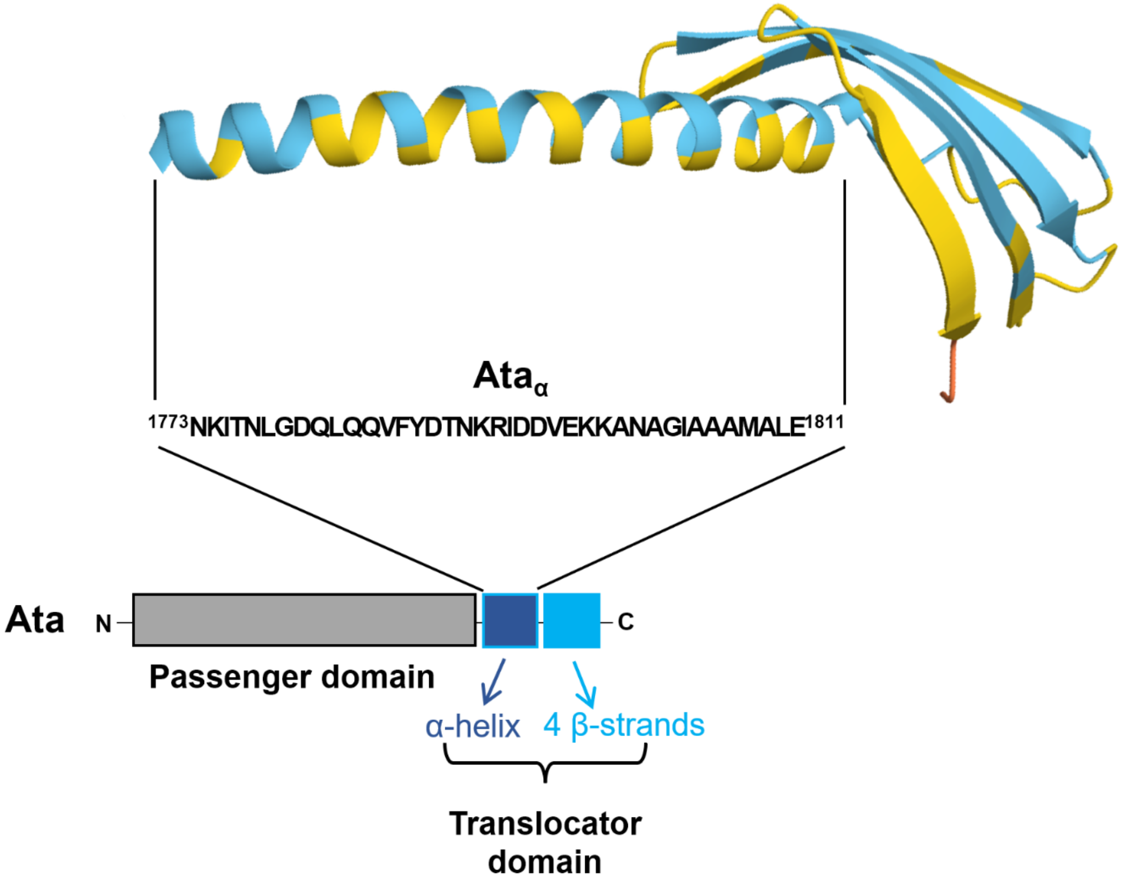

Supplementary Figure 1. Sequence of Ata$_{\alpha}$ and its position in Ata. The 3D structure of the C-terminal translocator domain of Ata was simulated by AlphaFold (https://alphafold.com/).

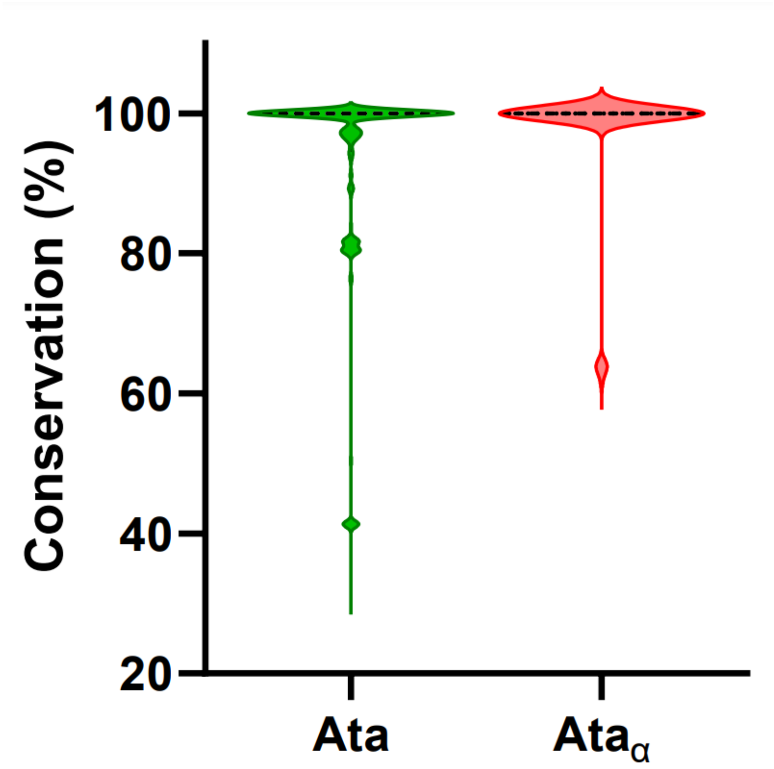

Supplementary Figure 2. Conservation analysis of Ata and Ata$_\alpha$. All protein sequence of Ata of A. baumannii in NCBI database were obtained by using "(trimeric autotransporter adhesin Ata [Protein Name] OR (trimeric [All Fields] AND autotransporter [All Fields] AND adhesin [All Fields] AND Ata [All Fields])) AND "Acinetobacter baumannii"[porgn]" parameter. Ata of 17978 strain was completely consistent with 56% (785/1393) of the searched sequences and Ata$_\alpha$ was 94% (1303/1393).

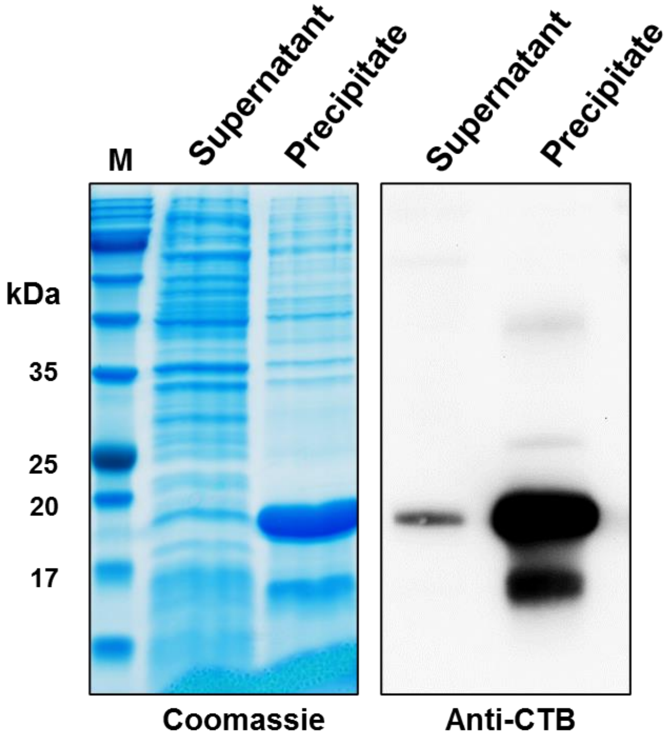

Supplementary Figure 3. Expression of CTB-Ata$_{\alpha}$. The recombinant plasmid pET30a-CTBAta$_{\alpha}$ was chemically transferred into *Escherichia coli* BL21(DE3). After adding 0.5 mM IPTG, the recombinant strain was cultured at 30°C for 12 h. Coomassie blue staining and Western blot were performed to analyze the expression of CTB-Ata$_{\alpha}$.

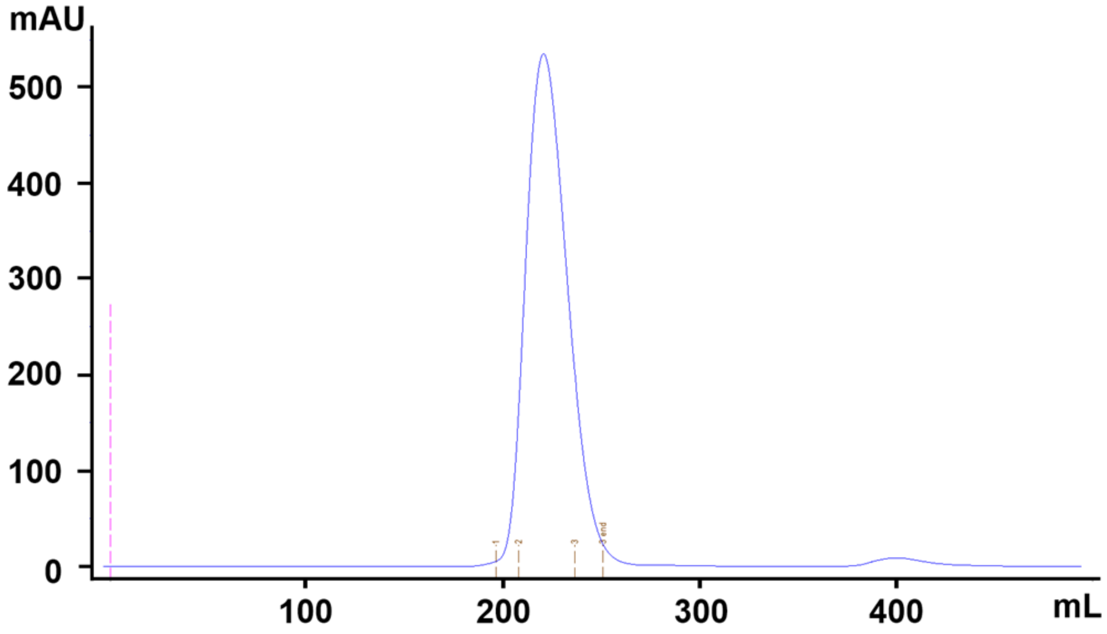

Supplementary Figure 4. Size exclusion chromatography elution profile (Superdex 75, φ 2.5 × 90 cm, 450 mL total volume, GE Healthcare) of CTB-Ata$_{\alpha}$. The retention volume of the peak was 220 mL.

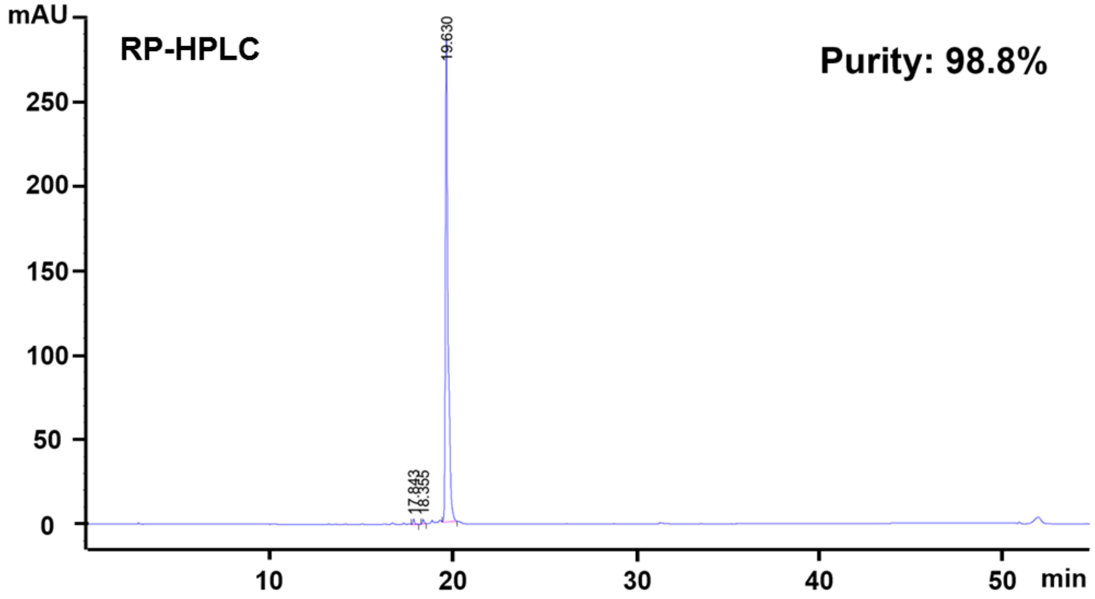

Supplementary Figure 5. Determination of the purity of CTB-Ata$_{\alpha}$ by RP-HPLC (Agilent ZORBAX 300SB-C8, 5μm, φ4.6×250mm) at 280nm. CTB-Ata$_{\alpha}$ was eluted with a gradient of 0 to 100% elution buffer (95% acetonitrile + 0.1% TFA) in 40 min.

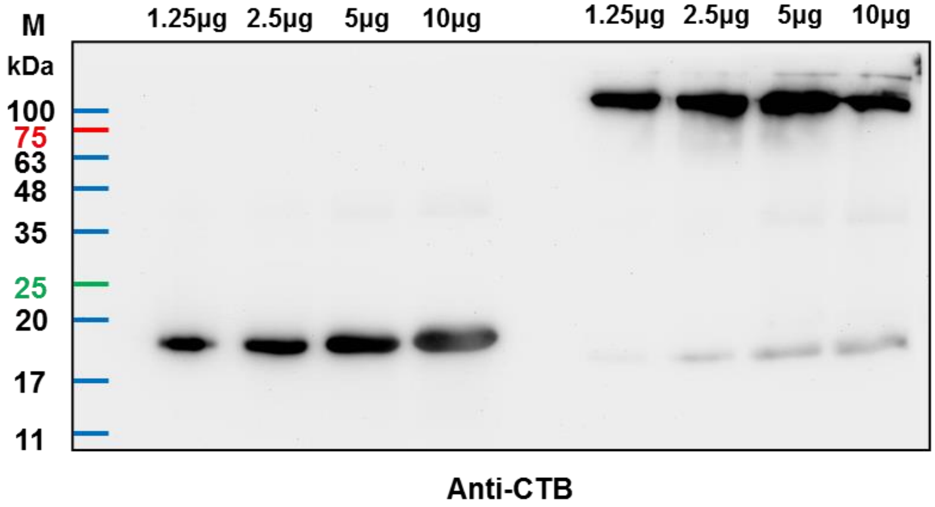

Supplementary Figure 6. Purified CTB-Ata$_{\alpha}$ was analyzed by Western blot with anti-CTB after reducing and non-reducing SDS-PAGE.

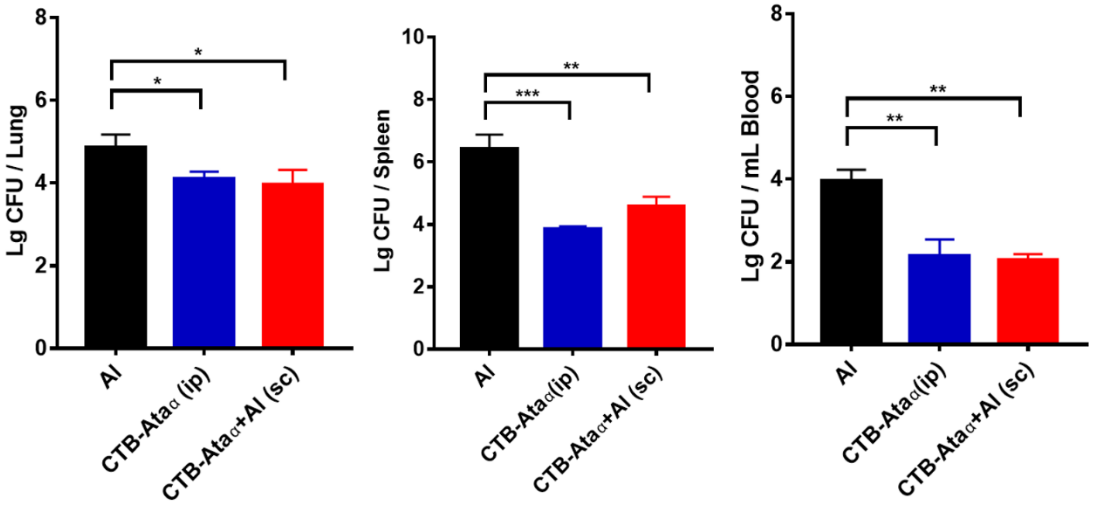

Supplementary Figure 7. Comparison of bacterial loads in lung, spleen, and blood between different immune pathway after sublethal challenge. Each group was compared using one-way ANOVA with Dunnett's multiple-comparison test: *P < 0.05, **P < 0.01.

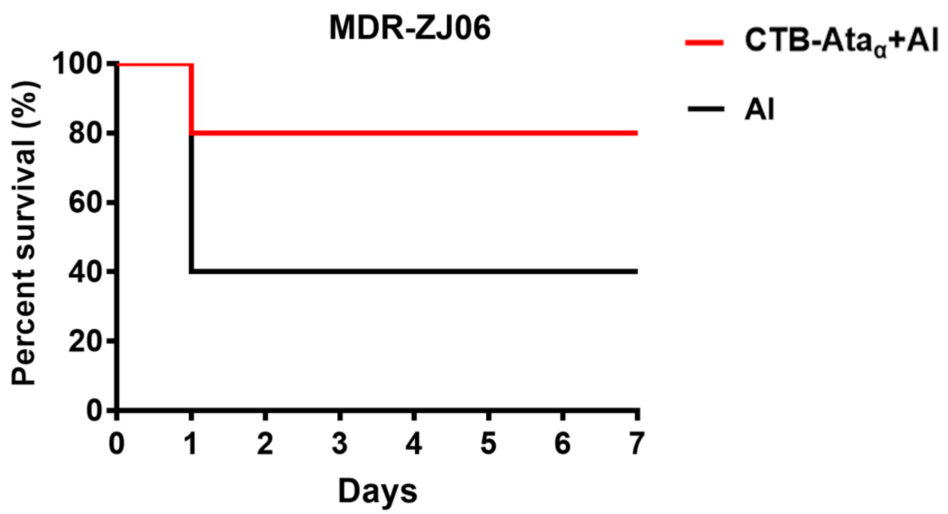

Supplementary Figure 8. Survival rates post-challenge with a lethal dose of MDR-ZJ006 (4.5 × 10^7 CFU) 14 days after the third immunization.

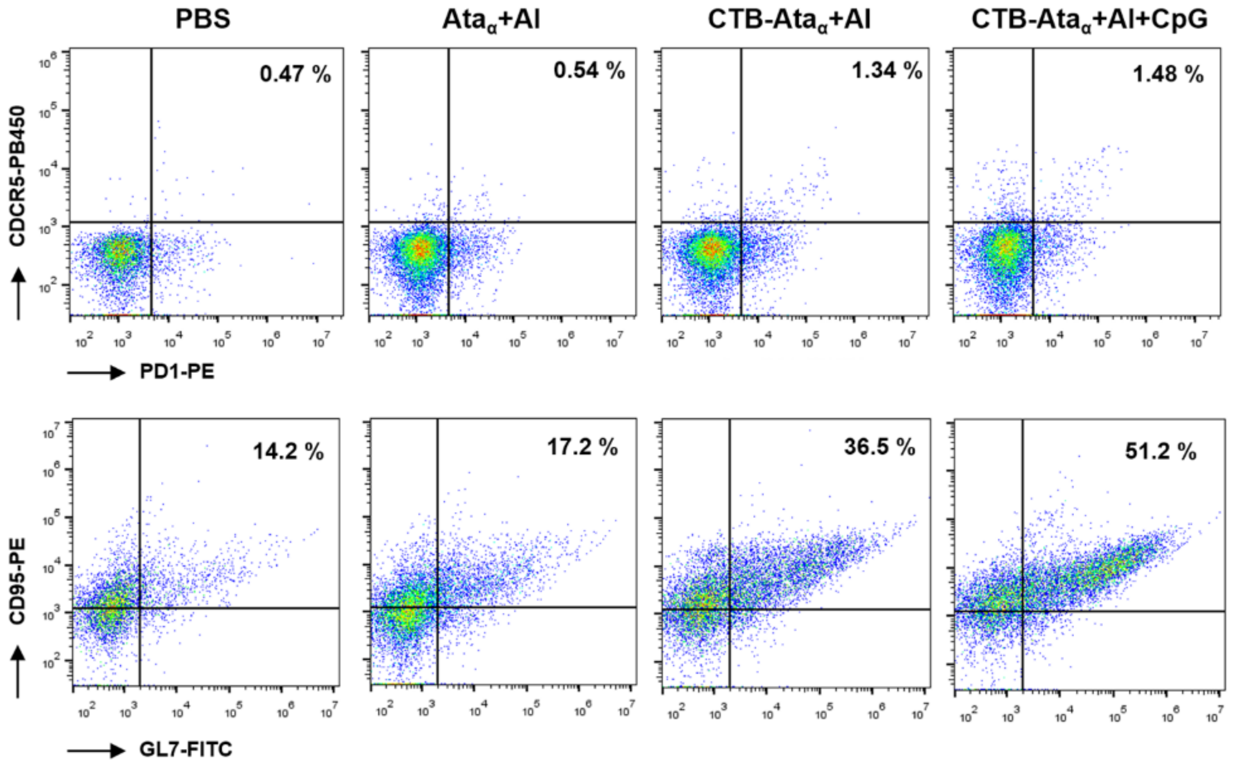

Supplementary Figure 9. Representative dot-plots of flow cytometry results. The proportion of Tfh cells ($CXCR5^+$ $PD\text{-}1^+$ among the $CD4^+$ cell population) and GC B cells ($GL7^+$ $CD95^+$ among the $B220^+$ cell population) in dLNs on day 7 postvaccination were detected by flow cytometry.

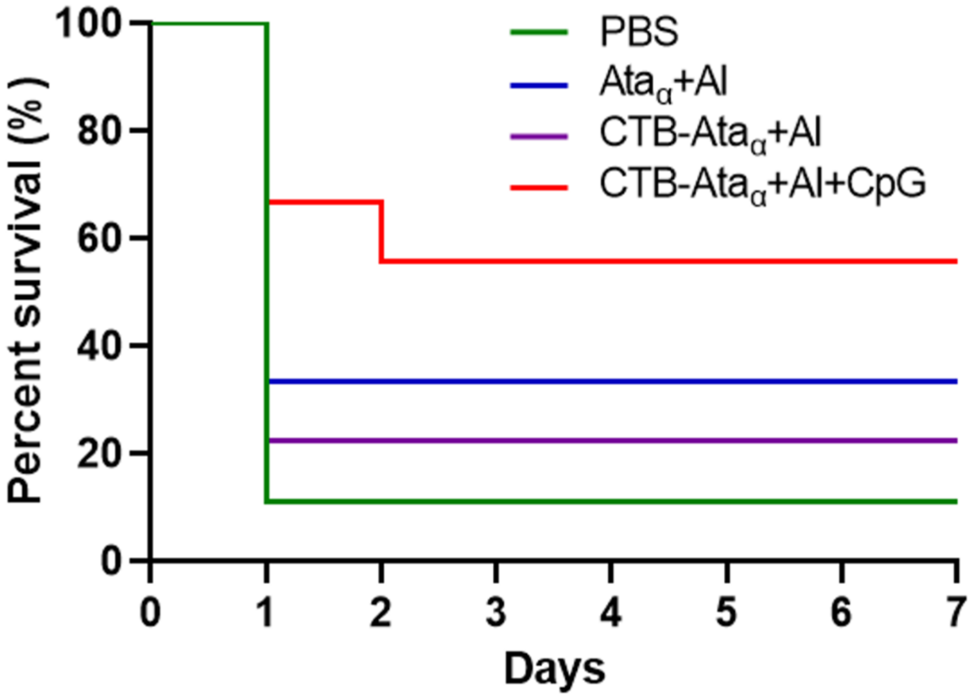

Supplementary Figure 10. Survival rates post-challenge with a lethal dose of ATCC 17978 (5.4 $\times$ 10^7 CFU) 14 days after the third immunization.
